# Maternal 3’UTRs: from egg to onset of zygotic transcription in Atlantic cod

**DOI:** 10.1186/1471-2164-13-443

**Published:** 2012-09-01

**Authors:** Lene Kleppe, Rolf B Edvardsen, Heiner Kuhl, Ketil Malde, Tomasz Furmanek, Øyvind Drivenes, Richard Reinhardt, Geir L Taranger, Anna Wargelius

**Affiliations:** 1 Institute of Marine Research, P. O. Box 1870, Nordnesgaten 50, 5817, Bergen, Norway; 2 Max Planck Institute for Molecular Genetics, Ihnestrasse 63-73, D-14195, Berlin-Dahlem, Germany; 3 Max-Planck Genome centre, MPI fuer Pflanzenzüchtungsforschung, Carl-von-Linné-Weg 10, D-80829, Koeln, Germany

**Keywords:** 3’UTR, Sequence/EST filtering, External spiking, Transcriptome, Teleost, Embryonic development

## Abstract

**Background:**

Zygotic transcription in fish embryos initiates around the time of gastrulation, and all prior development is initiated and controlled by maternally derived messenger RNAs. Atlantic cod egg and embryo viability is variable, and it is hypothesized that the early development depends upon the feature of these maternal RNAs. Both the length and the presence of specific motifs in the 3’UTR of maternal RNAs are believed to regulate expression and stability of the maternal transcripts. Therefore, the aim of this study was to characterize the overall composition and 3’UTR structure of the most common maternal RNAs found in cod eggs and pre-zygotic embryos.

**Results:**

22229 Sanger-sequences were obtained from 3’-end sequenced cDNA libraries prepared from oocyte, 1-2 cell, blastula and gastrula stages. Quantitative PCR revealed that EST copy number below 9 did not reflect the gene expression profile. Consequently genes represented by less than 9 ESTs were excluded from downstream analyses, in addition to sequences with low-quality gene hits. This provided 12764 EST sequences, encoding 257 unique genes, for further analysis. Mitochondrial transcripts accounted for 45.9-50.6% of the transcripts isolated from the maternal stages, but only 12.2% of those present at the onset of zygotic transcription. 3’UTR length was predicted in nuclear sequences with poly-A tail, which identified 191 3’UTRs. Their characteristics indicated a more complex regulation of transcripts that are abundant prior to the onset of zygotic transcription. Maternal and stable transcripts had longer 3’UTR (mean 187.1 and 208.8 bp) and more 3’UTR isoforms (45.7 and 34.6%) compared to zygotic transcripts, where 15.4% had 3’UTR isoforms and the mean 3’UTR length was 76 bp. Also, diversity and the amount of putative polyadenylation motifs were higher in both maternal and stable transcripts.

**Conclusions:**

We report on the most pronounced processes in the maternally transferred cod transcriptome. Maternal stages are characterized by a rich abundance of mitochondrial transcripts. Maternal and stable transcripts display longer 3'UTRs with more variation of both polyadenylation motifs and 3'UTR isoforms. These data suggest that cod eggs possess a complex array of maternal RNAs which likely act to tightly regulate early developmental processes in the newly fertilized egg.

## Background

A series of complex biological processes take place over time in order to transform a newly fertilized egg into a fully grown organism. At the very beginning of embryonic development, these biological processes are completely controlled by maternally derived materials. Maternally deposited messenger RNAs (maternal RNAs) and proteins drive embryo development until the zygote is able to transcribe its own RNA. The link between maternal mRNAs and the ability of the embryo to develop normally is of high relevance in many fields, including that of aquaculture. For aquaculture species including Atlantic cod (*Gadus morhua* L*.*), there is a necessity to produce viable eggs and embryos to ensure both economic- and welfare aspects. However, variable egg viability is common, especially in marine farmed fish species (Reviewed in [1,2]). Also, egg viability in cod varies between batches within one spawning season [3]. Although a number of studies have identified different factors that may affect egg viability, as well as indicators that could help in determining if an egg batch is of good or bad shape (reviewed in [4]), the complete picture is far from fully understood. During oogeneis transcribed maternal RNAs are stored in dormant complexes for translation just before or after fertilization [5]. The activation of translation is regulated and starts with polyadenlyation of the stored mRNA. A recent paper in zebrafish reports that 30% of maternal transcripts are polyadenylated prior to fertilization while 70% are progressively polyadenylated after fertilization [6]. In several vertebrates including *Xenopus*, zebrafish and mouse, elements (motifs) in the 3’UTR confer translation initiation and mRNA stability or degradation [5,7,8]. These 3’UTR elements bind specific proteins and/or protein-complexes which mediate stability, translation and degradation of maternal RNAs [5].

As development progresses, maternal transcripts gradually mix with zygotic transcripts and many are degraded, in line with the first events of embryogenesis. This maternal to zygotic transition (MZT) is characterized by a gradual degradation of maternal mRNAs and proteins, followed by transcriptional activation of the zygotic genome (reviewed in [9]). MZT coincides with cell cycle lengthening and loss of cell synchrony at cell cycle 13 in *Xenopus*, termed the midblastula transition (MBT) [10,11]. In zebrafish the MBT initiates at cycle 10 [12], when the embryo contains 512 cells [13]. In medaka MZT is uncoupled from the midblastula stage, and begins already at the 64 cells stage coinciding with the start of asynchronous cell division [14]. In contrast, a large proportion of the maternal RNA is degraded already at the 2-cell stage in mammals [15,16], highlighting the marked species-specific differences in the timing of mRNA degradation and subsequent zygotic genome activation.

**Figure 1:**
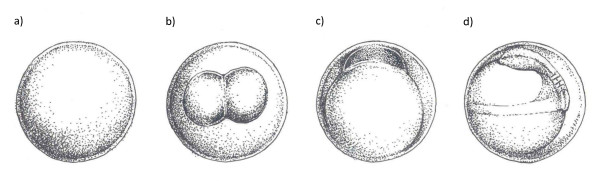
**The developmental stages of cod that were studied.** Drawing of the developmental stages of Atlantic cod that were used in this study; spawned, unfertilized egg (**a**), 1-2 cells (**b**), blastula (**c**) and gastrula (**d**). All oocytes referred to in this paper represent mature oocytes e.g. eggs.

The basic composition of maternal RNAs in the oocyte and early embryogenesis has only been assayed in a few teleost species including zebrafish [17,18] and recently Atlantic cod [19]. However, these studies looked at the composition of expressed genes using a microarray. Olsvik [20] characterized the blastula stage in cod, using a sequenced un-normalized cDNA library. This technique has also been applied to study oocytes and early stages of development in mouse, Atlantic halibut and zebrafish [6,21,22]. This type of study helps identifying overall abundance, novel transcripts, and splice variants in the transcriptome in contrast to microarray which only determine expression levels of a predefined subset of genes. Aanes [6] and Evsikov [21] did assay the 3’UTR sequence of the assayed maternal RNAs and were able to find regulatory signals conferring stability and function of maternal RNAs. Therefore the aim of this study was to characterize the composition of maternal RNAs in oocytes and early embryos of Atlantic cod, by sequencing 3’UTRs of RNA from unfertilized eggs and embryos of 1-2 cell-, blastula- and gastrula stage (Figure 1). *In silico* studies focus on transcript- and gene abundance, identity, diversity, ontology, stability and complexity in maternal vs. zygotic developmental stages. This work also focuses on two technical difficulties when analyzing this type of data; (1) the reliability of gene expression profiles obtained from cDNA libraries and (2) the difficulty of using internal reference genes when working with egg and early developmental stages. 

## Results and discussion

### Methodological considerations; number of sequences threshold and external spiking

**Figure 2:**
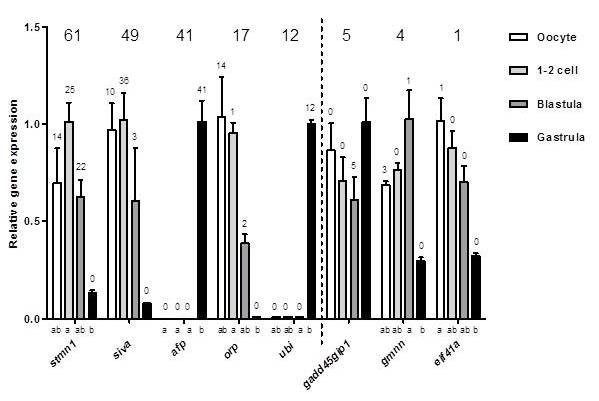
**Gene expression profiles measured with number of transcripts in libraries and qPCR.** Gene expression levels of *stathmin 1 (stmn 1), apoptosis regulatory protein siva (siva), antifreeze protein (afp), oogenesis related protein (orp), ubiquitin (ubi), growth arrest and DNA-damage-inducible, gamma interacting protein 1 (gadd45gip1), geminin (gmnn)* and *eif41a* (x-axis), relative to rabbit *alpha hemoglobin (hba)* (y-axis). The numbers above each bar represent the number of times the transcript was sequenced in that particular library. The bold number above each gene expression profile represents the total number of times the transcript was sequenced. The vertical dotted line indicates threshold for total n, in further library analysis. Different letters on x axis represent significantly different mRNA levels between stages for each gene. Genes with no letters on x-axis show no significant difference in gene expression between stages. All data are shown as mean with SEM. N = 3.

Transcriptome sequencing offers the possibility to discover novel expression patterns, high abundance transcripts, sequence motifs and splice variants [6]. In addition transcriptome sequencing measures abundance at a wider scale, as this technique does not get oversaturated. However challenges regarding quality do exist, it is for example crucial for the correct annotation to have a high-quality reference genome. Another aspect of quality is how reliable the expression patterns discovered in libraries really are. Highly expressed genes represent processes that dominate in the given developmental stage, and discovery and measurements of such gene expressions are more likely to be accurate than that of genes that are weakly expressed. Singletons (n = 1) are more likely to represent random sequencing or sequnecing errors than transcripts that are sequenced numerous of times for one gene. Likewise, in microarray studies, low number of transcripts causes a large variation in gene expression results [23]. This was also confirmed in this study as we revealed that for genes with few sequences (total n = 1-5) detected in the libraries, the expression profile from oocyte until gastrula could not be reflected in expression profiles measured with quantitative PCR (qPCR) for the same genes (Figure 2). For genes with a higher number (n = 12-61) of sequences, their expression profiles corresponded with the ones measured by qPCR (Figure 2). We therefore suggest for further transcriptomic studies to filter data based on analysis of number of transcripts to obtain reliable expression profiles for downstream analyses. A threshold of 9 or more total sequences for each gene was selected to be applied for our dataset, to be able to include only the most reliable data for analysis. Threshold 9 was chosen because it is in the middle of 5 and 12, and using this threshold did not considerably affect number of genes to be excluded for downstream analyses compared to when applying threshold 5. Given that only 1 EST was sequenced for approximately 50% of the genes in our dataset (data not shown), even a very low cutoff would exclude a high number of genes from the analysis. However some important information may be hidden within genes with low expression, and will therefore be lost by applying an EST number threshold. For example, the zebrafish oocyte possesses a considerable richness of maternally expressed genes of which many may be of importance for embryogenesis [6]. When the current experiment was carried out the cod genome was not yet sequenced. However, using the current genome [24] together with new sequencing technology could significantly increase number of reads analyzed in this study. For example, Aanes recently studied the zebrafish embryonic transcriptome by applying RNA sequencing [6], a method which provides a considerably more precise measurement of transcript abundance due to high number of sequence reads [20-22]. If we were to perform this study again with RNA sequencing, more sequence reads would be obtained and more genes could be included in the analysis as they would be represented by more than only 1 or a few sequenced transcripts. 

Also if candidate gene expressions from cDNA libraries are to be verified by qPCR, stable reference genes are essential. When it comes to early embryo tissue like in this study, such stable references are difficult to obtain. For example, neither *β-actinelongation factor 1- α (ef1α)18S, actin related protein (arp)* nor *ubiquitin (ubi)* showed stable reference gene expression in stages assayed for gene expression [Additional file 1, [25,26]. For this reason we chose not to use any of the reference genes tested, but instead apply a technique (external spiking) where external RNA (rabbit *alpha hemoglobin* (*hba*)) is added in equal amounts to each RNA sample [27]. The considerably more stable expression pattern of *hba* can be seen in [Additional file 1. We therefore suggest that external spiking may serve as a good alternative to the traditional reference genes previously applied in qPCR, especially when measuring gene expressions in early embryo tissue.

### Overall composition of cDNA libraries from oocyte, 1-2 cell, blastula and gastrula stage in Atlantic cod: amount of transcripts and genes, and mitochondrial contribution

In order to get an impression of the approximate timing of MZT (and therefore the shift from degrading to activating transcripts) we measured gene expression profiles of candidate genes known from other species to be maternal, zygotic or involved in events taking place at the initiation of MZT [Additional file 2. Our results together with the findings of Drivenes [19] indicate that the MZT takes place between blastula and gastrula in Atlantic cod, which is also true for zebrafish [12]. Together the cDNA libraries from oocyte, 1-2 cell, blastula and gastrula stage produced 26162 3’EST sequences, which have been added to GenBank; 6511, 6607, 6506 and 6538 sequences were obtained from oocyte, 1-2 cell, blastula and gastrula libraries, respectively. 22229 sequences (5538, 5875, 5238 and 5578 in oocyte, 1-2 cell, blastula and gastrula, respectively) were mapped to the Atlantic cod genome [24]. 17025 of these sequences hit cod genes with the rules given in material and methods section; they were predicted to encode 2611 genes, of which 2051 (78.6%) were Ensembl-annotated. 23 genes were pseudogenes, and were therefore removed from most of the analysis. Furthermore as described in methodological considerations, sequences with n ≤ 8 were removed from most of the analysis, leaving us with 12764 sequences predicted to be encoded by 257 genes. Compared to similar analyses [20-22], 257 genes is a low number; however these studies have included most transcripts in contrast to the current study. As mentioned, we showed no correlation between qPCR and low copy number of ESTs (N < 9, see Figure 2 and discussion first section; methodological considerations), and highly expressed genes (N > 9) are possible to classify as maternal, zygotic, degrading and activating. EST copy number filtering was a necessity to be able to study stability and complexity of dominating transcripts in early embryos of cod.

**Figure 3:**
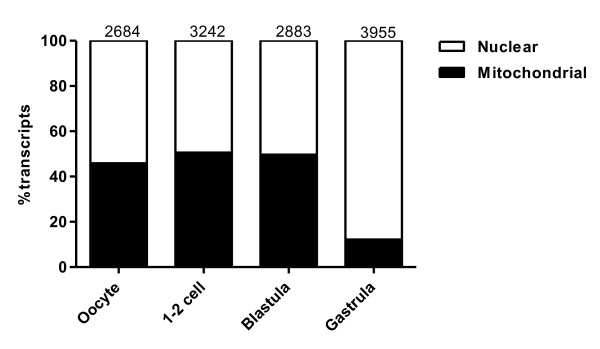
**Distribution of mitochondrial transcripts.** Overview of the proportion of mitochondrial transcripts (y-axis) in the oocyte-, 1-2 cell-, blastula- and gastrula libraries (x-axis). The numbers on top of each bar represent total number of transcripts for that particular stage/library.

Strikingly, the overall composition of abundant transcripts obtained from maternal stages (oocyte, 1-2 cell and blastula) contained 45.9-50.6% mitochondrial transcripts, while at the zygotic stage (gastrula) only 12.2% of the transcripts were mitochondrial (Figure 3). Less than 5.1% of all the expressed genes in each developmental stage encoded these numerous mitochondrial transcripts (data not shown). This trend was also evident by the highly expressed mitochondrial genes of Table 1, showing the 20 most abundantly expressed genes when all transcripts from all libraries were included. Likewise it has been reported a high level of mitochondrial transcripts at the 2-cell stage in halibut [22] and in the oocyte of Senegalese sole [28]. High level of mitochondrial transcripts in mature oocytes may be linked to high metabolic need to complete synchronous continuous cell-divisions at early developmental stages, whereas at later stages the energy metabolism is more localized to target areas in specific development. It has even been suggested that a major part of maternal RNAs in egg-laying organisms lack specificity and only provides supplemental nutrition [29]. However a proportion of the maternal RNAs are essential for normal development in fish, as several studies report abnormal embryo development when mRNA translation is inhibited [30]. Also several processes in zebrafish morphogenesis, most notably brain formation, fail to occur normally when normal degradation of maternal RNA is disturbed [31]. 

**Table 1:** The 20 most abundant transcripts

**Gene**	**Total**	**Oocyte**	**1-2 cell**	**Blastula**	**Gastrula**
Mitochondrial genes					
*Cytochrome c oxidase subunit II*	3418	910	1293	881	334
*Cytochrome B*	563	137	114	263	49
*Cytochrome c oxidase subunit I*	353	90	110	119	34
*ATP synthase F0 subunit 8*	176	36	45	84	11
*ATP synthase F0 subunit 6*	144	32	45	50	17
Nuclear genes					
*H2A Histone family, member V*	463	94	208	82	79
*Tetraspanin 3*	185	3	20	159	3
*Small nuclear ribonucleoprotein polypeptide E*	133	25	34	7	67
*Ribosomal protein S20*	128	2	1	2	123
*Ribosomal protein S12*	111	7	1	1	102
*Ribosomal protein S23*	106	0	0	0	106
*Ribosomal protein L34*	103	4	4	16	79
*CDKN2A interacting protein N-terminal like*	103	27	28	19	29
*COX17 cytochrome c oxidase assembly homolog*	101	60	28	13	0
*Unknown*	97	46	48	2	1
*Small nuclear ribonucleoprotein D3 polypeptide 18kDa*	95	16	36	32	11
*Small nuclear ribonucleoprotein polypeptide F*	84	10	8	63	3
*Mago-nashi homolog B*	78	10	25	39	4
*Unknown*	78	0	0	0	78
*Unknown*	74	2	0	3	69

Gene names and their total number of transcripts in oocyte, 1-2 cell, blastula and gastrula are shown.

### Nuclear transcripts and expressed genes: distribution and gene ontologies

**Figure 4:**
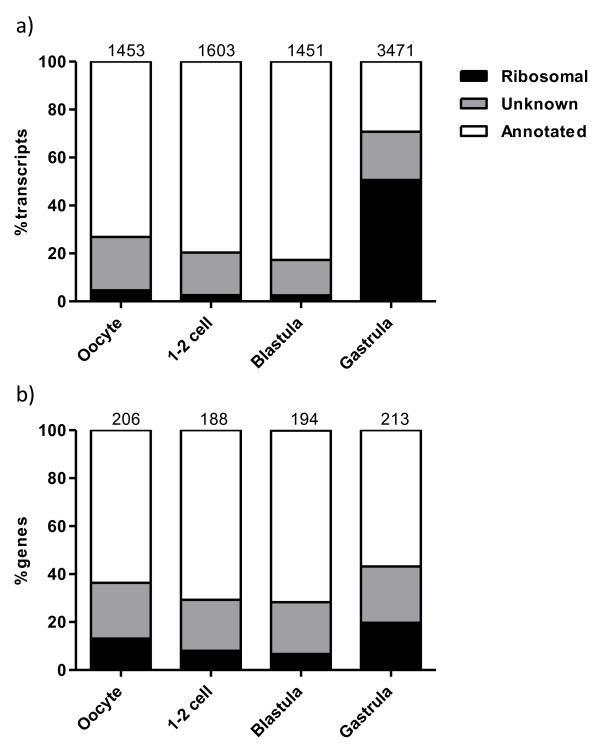
**Distribution of nuclear transcripts and the genes encoding them.** Overview of the distribution (y-axis) of nuclear transcripts (**a**) and the genes encoding them (**b**) in the oocyte-, 1-2 cell-, blastula- and gastrula libraries (x-axis). The numbers on top of each bar represent total number of transcripts or genes for that particular stage/library.

To facilitate analysis of expressed genes from the genome, mitochondrial transcripts (4786, encoded by 10 genes) were excluded. What remained were the nuclear transcripts (7978, encoded by 247 genes). A significant feature of the gastrula nuclear transcriptome was a high presence of ribosomal transcripts (50.6%) compared to the earlier stages (2.5–4.6%, Figure 4a). This increase in transcript abundance was not a consequence from many additional genes being expressed in the gastrula stage, as the number of genes encoding the ribosomal transcripts only increased from 6.7–13.1% in the maternal libraries to 19.7% in the gastrula library (Figure 4b). However, some of the ribosomal transcripts were uniquely found in the gastrula library (Table 2). A similar pattern of ribosomal transcripts has previously been found in zebrafish [17] and halibut [22]. The high presence of ribosomal transcripts in gastrula is most likely due to major onset of transcription since ribosomal proteins are required for the production of ribosomes at the site for transcription. 

**Table 2:** The 17 transcripts present in the gastrula library only

**Gene**	**Total**	**Oocyte**	**1-2 cell**	**Blastula**	**Gastrula**
Nuclear genes					
*Ribosomal protein S23*	106	0	0	0	106
*Unknown*	78	0	0	0	78
*Ribosomal protein L27a*	58	0	0	0	58
*Ribosomal protein L28*	54	0	0	0	54
*Unknown*	50	0	0	0	50
*Ribosomal protein L18a*	48	0	0	0	48
*Ribosomal protein L24*	48	0	0	0	48
*ribosomal protein S27*	48	0	0	0	48
*Ribosomal protein S19*	46	0	0	0	46
*Unknown*	41	0	0	0	41
*Unknown*	40	0	0	0	40
*Ribosomal protein L31*	36	0	0	0	36
*Ribosomal protein S7*	30	0	0	0	30
*Ribosomal protein, large, P2*	26	0	0	0	26
*Ribosomal protein, large, P1*	14	0	0	0	14
*Ubiquitin A-52 residue ribosomal protein fusion product 1*	12	0	0	0	12
*Small nucleolar RNA, C/D box 68*	9	0	0	0	9

Gene names and their total number of transcripts in oocyte, 1-2 cell, blastula and gastrula are shown.

Most nuclear genes (79.4%) were expressed in more than one developmental stage, and 51.8% (128) of the genes were expressed in all four stages. The most highly expressed genes (see Table 1) present in all libraries were *histone H2A family member V* followed by *tetraspanin 3,* which are involved in general processes like structuring of chromosomal fibres and signal transduction in cell development, activation, growth and motility. 12.1% (30) of the nuclear genes were expressed exclusively in maternal stages (oocyte only or oocyte and one or two of the next developmental stages), and represented a diverse group (Table 3). However among the ones with the highest numbers of transcripts in this category, we found common genes (like *COX17stathmin* and cyclins) involved in processes like energy metabolism and cell cycle, which are features that characterize early embryonic development. Other studies [6,22,32] report on a higher number of strictly maternal genes than 30, however as mentioned many genes were excluded in this study due to their low number of sequenced ESTs. Since the cutoff of N = 9 may have excluded many lower expressed but important maternal genes from downstream analysis all sequenced genes were added to GenBank, leaving them available for future studies of for example egg viability. 

**Table 3:** The 20 most abundant transcripts found in maternal libraries only

**Gene**	**Total**	**Oocyte**	**1-2 cell**	**Blastula**	**Gastrula**
Nuclear genes					
*COX17 Cytochrome c oxidase assembly homolog*	101	60	28	13	0
*Stathmin 1*	61	14	25	22	0
*Adenylosuccinate synthase like 1*	49	10	36	3	0
*S100 calcium binding protein A1*	41	9	26	6	0
*THAP domain containing 4*	34	10	16	8	0
*Unknown*	25	7	0	18	0
*Uncharacterized protein C14orf119*	24	0	1	23	0
*Unknown*	20	6	9	5	0
*Claudin 4*	20	9	6	5	0
*Unknown*	20	8	4	8	0
*LSM12 homolog*	19	3	15	1	0
*UPF0139 membrane protein C19orf56*	17	2	11	4	0
*Topoisomerase (DNA) I*	17	10	5	2	0
*Unknown*	17	7	4	6	0
*Unknown*	17	14	1	2	0
*Cyclin A1*	15	2	6	7	0
*Protein (peptidylprolyl cis/trans isomerase) NIMA-interacting, 4 (parvulin)*	15	5	2	8	0
*Cyclin A2*	15	3	1	11	0
*H1 histone family, member O, oocyte-specific*	14	5	3	6	0
*Unknown*	12	4	7	1	0

Gene names and their total number of transcripts in oocyte, 1-2 cell, blastula and gastrula are shown.

We also checked how the nuclear transcripts were distributed when including all nuclear genes (2575) without any threshold for number of transcripts. In this dataset a large proportion (56.5%) of the genes had only 1 transcript. Consequently, the genes expressed exclusively in maternal and gastrula stages showed an increase in their proportions (12.1–31.9% and 6.9–13%, respectively). The proportion of genes expressed in all developmental stages decreased markedly from 51.8 to 6.5% [Additional file 3]. It is therefore clear that the use of a threshold for number of transcripts makes a difference for describing gene expression patterns.

Functional profiles of the most common nuclear transcripts in oocyte, 1-2 cell, blastula and gastrula (obtained from the use of gene ontology (GO)-terms) revealed that both within cellular component, molecular function and biological process many of the transcripts did not change much in their abundance over time (data not shown). However some differences could be observed between maternal and zygotic stages [Additional file 4. An increase in proportion of transcripts that were part of macromolecular complexes from maternal to zygotic developmental stage possibly reflects specification of cell types which requires more complex molecules as the embryo enters the zygotic stage. At the molecular function level, a pronounced increase of transcript amount from maternal to zygotic stage was evident for structural molecule activity, reflecting a shift towards specification of cell function. Several biological processes including cellular component organization or biogenesis, multicellular organismal process and developmental process showed a marked shift between maternal and zygotic stage, with increase in transcript proportion in gastrula. Taken together, these changes reflect more specialized and complex processes developing after MZT in cod, as would be expected since gastrula is a developmental stage where intricate processes like germ layer formation takes place (reviewed in [33]).

### 3’UTR structure: isoforms, length and motifs

Based on manual annotation, 3’UTR length was predicted for all sequences with stop codon and poly-A tail (in total 191 genes). To be able to study the gene expression dynamics over time from a maternal to a zygotic embryo, genes (when possible) were strictly classified as having degrading, stable or activating transcripts. In order to be termed degrading, minimum 95% of the transcripts had to be located in maternal stages (oocyte until blastula). In order to be termed activating, minimum 95% of the transcripts had to be present in blastula and gastrula. Transcripts present in all 4 stages, with a distribution of minimum 10% and maximum 40% in one stage were termed stable. Applying this classification gave 35, 26 and 39 genes with degrading, stable and activating expression profiles, respectively. 91 genes could not be classified according to these specifications. To ensure reliable results, the following analyses on 3’UTR structure were also performed when applying less strict specifications [Additional file 5].

**Figure 5:**
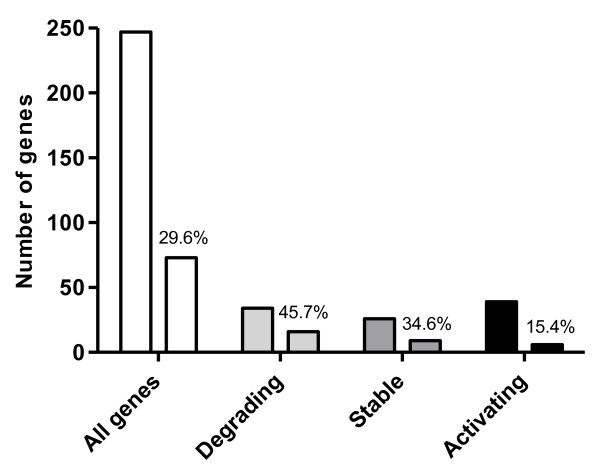
**3’UTR isoforms.** Overview of number of genes with 3’UTR isoforms (y-axis) within the whole dataset, and within genes with degrading, stable and activating transcripts (x-axis). The bar to the left within each category represents the total number of genes for that category, and the bar to the right represents the corresponding number of genes with 3’UTR isoforms. Percentages of genes with 3’UTR isoforms within each group are indicated by the number on top of the bars.

Totally 73 nuclear genes (29.6%) expressed more than one 3’UTR isoform. Among genes with degrading and stable transcripts a relatively high amount (45.7 and 34.6%, respecively) displayed 3’UTR isoforms (Figure 5). Alternatively spliced transcripts are common in early embryos in other species, for example in zebrafish where it has been shown 50–60% alternative splicing of transcripts [6]. Also in mouse embryos a significant proportion of alternatively spliced transcripts have been found [34], and in this context it was suggested that alternative splicing is of importance in the regulation of development. Activating genes of cod had a far less degree of 3’UTR isoforms (15.4%, Figure 5). This may be an indication that alternative splicing is a less important regulatory mechanism for genes that are more active in early zygotic stages, than for genes that are more active in maternal stages. 

**Figure 6:**
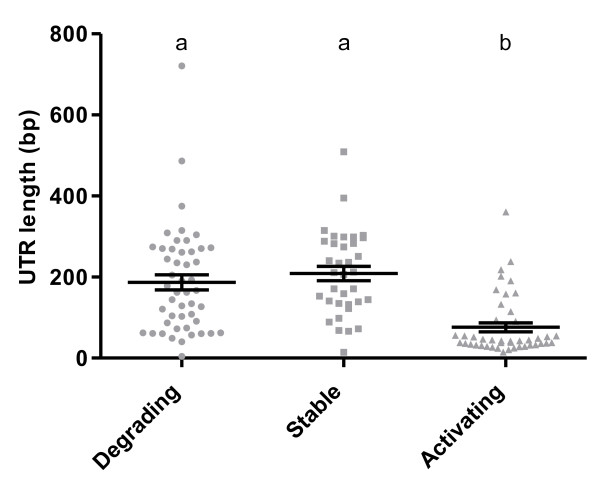
**3’UTR length.** 3’UTR-lengths of degrading, stable and activating transcripts. Different letters represent significant difference in 3’UTR length. All data are shown as mean with SEM. N = 48, 35 and 45 for degrading, stable and activating transcripts, respectively.

Mean 3’UTR length (when including all isoforms) in degrading (n = 48), stable (n = 35) and activating (n = 45) transcripts was 187.1, 208.8 and 76 bp, respectively (Figure 6). Activating transcripts had significantly (p < 0.0001) shorter 3’UTR length than degrading and stable ones. A higher proportion of long maternal 3’UTRs have previously been found across the animal kingdom [29] associated with lower turnover-rate of RNAs [35]. Our findings suggest that also in cod, transcripts from maternally active genes have longer 3’ UTRs. The shorter 3’ UTRs of the activating transcripts may also be explained by the fact that many of them were ribosomal (data not shown); such transcripts have been shown to contain short 3’UTRs [36]. Excluding ribosomal genes would lead to a small dataset. Consequently, we applied less strict classification criteria for degrading, stable and activating transcripts and found that also non-ribosomal activating transcripts have significantly shorter 3’UTRs [Additional file 5. 

Although not statistically proved in this study, stable transcripts appeared to have the longest 3’UTRs (Figure 6). This corresponds to the suggestion that more stable RNAs have longer UTRs [37]. A strong exponential correlation has been found between 3’UTR length and morphological complexity (number of cell types in the organism) [38]. Increased regulatory complexity in long 3’UTRs may be a result of more miRNA binding sites. Furthermore, additional polyadenylation signals may produce transcript isoforms with several combinations of miRNA binding sites, which adds even more complexity [39]. It was observed an enrichment of 3’UTR isoforms in degrading and stable transcripts (abundant in the maternal stages) compared to activating ones (abundant in zygotic stage). This result suggests a more complex post-transcriptional regulation in the maternal stages since transcriptional regulation cannot be applied to control developmental processes prior to MZT.

**Figure 7:**
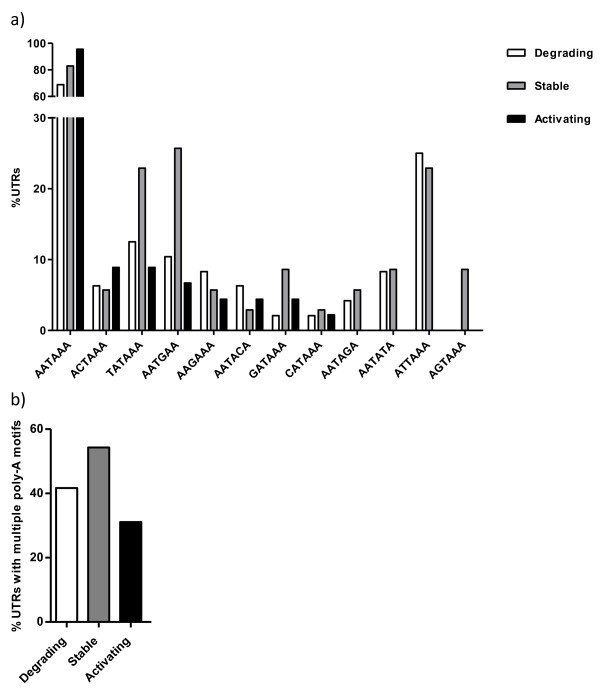
**Polyadenylation motifs.** Proportion (%) of degrading, stable and activating transcripts that contain different polyadenylation motifs (AATAAA, ATTAAA, AGTAAA, TATAAA, CATAAA, GATAAA, AATATA, AATACA, AATAGA, ACTAAA, AAGAAA and AATGAA) (**a**) and more than one of the motifs shown in **a**) (**b**). N = 48, 35 and 45 for degrading, stable and activating transcripts, respectively.

The apparent complex features of transcripts that were abundant in early developmental stages of cod were further investigated by searching for motifs in the 3’UTRs of degrading, stable and activating transcripts. Using Multiple Em for Motif Elicitation (MEME) [40] the presence of the polyadenylation motif AATAAA was indicated. Manual inspection of the sequences revealed that AATAAA (the exact motif) was present at an increasing manner from degrading (68.8%), through stable (82.9%) and to activating (95.6%) transcripts (Figure 7a). AATAAA is essential for cytoplasmic polyadenylation to take place [41,42]; that is, cleavage followed by adding of a poly-A tail to the 3’ end of the mRNA which then activates translation. Therefore polyadenylation is a key process in regulation of translation (for recent reviews see [43,44]), and thus serves as an important step in post-transcriptional regulation in maternal stage embryos. Also, it was recently found that cytoplasmic polyadenylation of maternal RNAs prior to MBT is essential for normal morphogenesis post-MBT in zebrafish [6]. It is known that AATAAA is found in nearly all known mRNAs, and that it is highly conserved. Our finding that 31.2 and 17.1% of degrading and stable transcripts (with known 3’UTR length) did not contain AATAAA may be explained by the possibility of transcripts to contain alternative polyadenylation motifs, where AATAAA have been slightly modified (reviewed in [43]). Therefore, we investigated the presence of 11 other putative alternative polyadenylation motifs [45]; for each sequence we observed which motifs were present, and if there was more than one motif (Figure 7). 3 motifs (ATTAAA, AATATA and AATAGA) were found exclusively in degrading and stable transcripts, and AGTAAA was present only in stable transcripts. None of the motifs were unique for activating transcripts, and most of the motifs were present at the lowest rate in this group of transcripts (Figure 7a). Also, degrading and especially stable transcripts had a higher prevalence (41.7 and 54.3%, respectively) of multiple motifs (more than one of the motifs studied) than activating ones (31.1%, Figure 7b). A higher degree (diversity and quantity) of putative alternative polyadenylation motifs in maternal degrading and stable transcripts suggests that these transcripts may apply alternative polyadenylation more frequently than activating transcripts. Polyadenylation at different sites produces different variants of the 3’UTR, which contributes to a more complex transcriptome [46]. 

## Conclusions

This study shows that scarce transcripts represent a significant bias in transcriptomic assessments of sequenced libraries, and suggests for future studies to compare number of transcripts and qPCR analysis to set a threshold for the transcript abundance that reflects reliable expression profiles. This study also shows a solution for dealing with the problem of internal variation in housekeeping genes when analyzing relative expression over time in fish embryos. By adding an external normalization gene the problem with housekeeping gene variation was avoided [27].

In maternal stages (oocyte to blastula), mitochondrial genes encoded 45.9–50.6% of the transcripts, reflecting a high energy demand in embryos up until MZT. The zygotic nuclear transcriptome had a high presence of ribosomal transcripts (50.6%), reflecting a major onset of transcription at this stage. Gene ontology terms reflect a more complex and specialized gastrula compared to maternal developmental stages. Degrading and stable transcripts have longer 3’UTRs than the ones that increased in abundance after MZT. Furthermore, this group of transcripts has more (diversity and amount) of the alternative polyadenylation motifs studied, as well as a higher number of 3’UTR isoforms. Together this indicates a more complex post-transcriptional regulation for genes that are highly active in maternal stages compared to that of genes that are more active in the zygotic embryo of cod.

In summary, this study describes in detail and for the first time, the overall level and composition of common maternal RNAs in the mature cod egg and early stages of embryogenesis, and reveals a picture of the fluctuations in the high abundance transcripts from egg to maternal stages and onwards to onset of zygotic transcription. This characterization of the maternal transcriptome in Atlantic cod (*Gadus Morhua* L.) will be helpful in further studies with the aim to elucidate the relationship between maternal RNAs and offspring viability in this species.

## Methods

### Egg and embryo collection

Eggs and sperm from Atlantic cod were obtained from Parisvatnet Research station, Institute of Marine Research, Øygarden, Norway in April 2009. Eggs were manually stripped from one female and sperm was collected from one male. Before freezing of unfertilized eggs, egg fluid was carefully aspirated and subsequently eggs were flash frozen liquid nitrogen. Remaining eggs were fertilized with the collected sperm and incubated at 7°C until they reached 1-2 cells, blastula and gastrula stages (Figure 1), embryos were staged according to Hall [47]. At sampling, staged embryos were collected and excess water was aspirated off before embryos were flash-frozen on liquid nitrogen. All collected samples were stored at −80°C until RNA isolation was initiated. All egg and embryo samplings were done according to guidelines approved by the Norwegian Animal Research Authority (NARA).

### cDNA library construction

From eggs and each developmental stage, total RNA was isolated using iPrep™ Trizol® Plus RNA Kit (Invitrogen, Carlsbad, CA, USA). The quality of RNA showed RIN values above 9.5 for all samples used (Bioanalyzer 2100, Agilent, Santa Clara CA, United States). cDNA libraries were constructed from 1.0 μg of RNA from each sample using Creator SMART cDNA Library Construction Kit (Clontech Laboratories, Inc., Mountain View, CA, USA). The cDNA was size-selected for 500bp according to Evsikov [21]. Transformation of the ligation mix was performed using the DH5αMAX competent cells (Invitrogen, Carlsbad, CA, USA). The titers of libraries used were for oocytes 7,7x10^5^, 1-2 cell stage 4,7x10^5^, blastula 1,5x10^6^ and gastrula 1,4x10^6^ clones per μl of ligation mix. The clones were grown on robot plates (Nalgene Nunc International, Rochester, NY, USA) filled with Luria-Bertani (LB) agar and chloramphenicol (30mg/L). Positive clones were randomly selected by automated picking robots and transfered to 384-well plates containing HMFM freezing media. Culturing was performed for 16h at 37°C without agitation. For isolation and purification of plasmid DNA 384-deepwell plates containing 190μl 2YT media and 30 mg/L chloramphenicol were inoculated from the freezing stocks. Clones were grown for 16-18h at 1100 rpm and 37°C. Isolation and purification of plasmids was done by an automated system, applying alkaline lysis followed by size selective DNA precipitation in PEG/2-Propanol [48]. Quantified plasmids (UV photometry) were sequenced using T7 primers following the Big-Dye version 3.1 protocol and capillary gelelectrophoresis by an ABI 3730xl system (Applied Biosystems Inc., Foster City, Ca, USA) at Max Planck Institute for Molecular Genetics, Berlin, Germany.

### Bioinformatical analysis of raw data

Sequence trace files from each library were processed employing the Phred base-calling algorithm [49,50]. Quality trimming and removal of contaminating vector sequences were performed using cross match and RBR [51] with the Uinivec filtering database from the National Center for Biotechnology Information (NCBI). Clustering and assembly were carried out using the TIGR Gene Indices Clustering tools (TGICL) [52]. Sequences for the libraries were added to GenBank; oocyte library [GenBank: JK777860-JK784370], 1-2 cell library [GenBank: JK771253-JK777859], blastula library [GenBank: JK758209-JK764714] and gastrula library [GenBank: JK764715-JK771252], respectively.

Clustered sequences were assembled into contigs (Fasta sequences are given in [Additional file 6) which were further annotated with Ensembl predicted genes [53] by mapping the contigs to the Atlantic cod genome [24] with a threshold above 75% identity BLAT hit against a genome scaffold in a gene or within 2000 bp downstream of a gene. Sequences that passed the following rules were included in further analysis: 1) above 85% identity BLAT hit against a genome mapped EST-contig, or 2) above 75% identity BLAT hit against a genome scaffold in a gene or within 2000 bp downstream of a gene. The mapping of the contigs can be seen in [Additional file 7.

Functional profiles of transcripts were constructed by applying GO-terms to group the transcripts [54]. The Ensembl genes were GO-annotated using BLAST against SwissProt and UniRef90 and annotation files from [55]. BLAST and GO files for SwissProt and UniRef90 that are constructed for Ensembl-annotated genome assembly can be seen in [Additional file 8 and [Additional file 9, respectively.

### Quantitative PCR

Total RNA was extracted by applying RNeasy® Mini Kit (QIAGEN, Oslo, Norway), and genomic DNA was removed by using Turbo DNA-free^TM^ Kit (Ambion, Austin, Texas, USA), according to the manufacturers instruction. Additionally, all samples were precipitated and redisolved in ddH20 after DNAse treatment. A NanoDrop® NP-1000 spectrophotometer (NanoDrop technologies, Wilmington, DE, USA) was used to measure the quantity and quality of the RNA samples. Samples with a 260/280 nm absorbance ratio outside the range 1.8–2.1 were excluded for further analysis. 1 pg of rabbit *hba* mRNA (SIGMA; Norway) was added to each RNA sample before cDNA synthesis (spiking). cDNA was produced using Superscript VILO cDNA synthesis (Invitrogen, Carlsbad, Germany). All primers used for amplification and detection of genes were designed applying the software Primer Express 3.0 (Applied Biosystems, Foster city, CA, USA), and are listed in Table 4 together with *hba*-primers [27]. Quantitative PCR (qPCR) was performed on an SDS 7900HT Fast Real-Time PCR system (Applied Biosystems, Oslo, Norway) system using SYBR® green PCR Master Mix (Applied Biosystems, Foster city, CA, USA), and the thermal cycling conditions were: 50°C for 2 min followed by 98°C for 10 min, and 40 cycles of 95°C for 15s followed by 60°C for 1 min. PCR efficiencies were verified to be equal between target and calibrator genes (Standard-curve method using 250, 125, 62.5, 31.25 and 15.625 ng RNA). Melting curve analysis was performed to verify presence of only one PCR product. 62.5 ng RNA was used to produce cDNA used for downstream analysis of relative abundance of transcript. For each PCR plate, no-template controls were run for each gene. The relative gene expression level was calculated using the Comparative Ct method (Applied Biosystems). All data are normalized to *hba* mRNA (spike), and the data for each gene are calibrated to its lowest mean ΔCt. 

**Table 4:** Gene short names, gene/EST ID and PCR primer sequences (5’-3’)

**Gene**	**Gene ID**	**5’-3’ forward primer sequence**	**5’-3’ reverse primer sequence**
*hba*	NM_001082389.2	GCAGCCACGGTGGCGAGTAT	GTGGGACAGGAGCTTGAAAT
*eif41a*	ENSGMOG00000012412	GACGATTGAGTCGATAGTGAATGC	CGCCCACCTGTTCTGTAAGG
*tbp*	ENSGMOG00000012435	CACAGCTGCAGAACATTGTATCAA	TGACGGCCGCAAAACG
*t*	ENSGMOG00000017391	CAACGAGATGATCGTCACCA	TGGCTGCTGATCATCTTCTG
*orp*	JK781166.1	GCTTCGGCTCCTTTTTATTTTTT	CCACTTGTTGTTCCGCAGAGT
*gmnn*	ENSGMOG00000008149	GATGGTATCTCAAATGAAGCCTATGA	CGCTCGTCTGCCACTTCTTT
*stmn1*	ENSGMOG00000005322	GAAACTATTGGAGATATTCAGGTTAAGGA	GCAGGAGCAGCCAGAATCAC
*siva*	JK777503.1	GAAGCACAGCCTTCTCAACCA	CCAATGAGCGTTTGTCCCTTT
*gadd45gip1*	ENSGMOG00000012967	CCGCTGAAACTGAACCTCAAG	GCCATTCGGGCGTCTTCT
*afp*	ENSGMOG00000002484	GAGCTGATGGCGACTGTTCA	CCTCCCGTCCTCGATGATG

When possible, sequences are listed with an Ensembl gene ID. Otherwise the sequences are listed with GenBank EST ID (*orp and siva*) or mRNA ID (*hba*).

### Statistical analyses

All data to be analyzed for significant differences were initially tested for Gaussian distribution by applying a D’Agostino-Pearson normality (omnibus K2) test. All mRNA levels quantified by qPCR had too few replicates (n = 3) for each developmental stage in order for the test to reveal normality or not. Therefore a non-parametric Kruskal-Wallis test was applied to reveal if the genes were differentially expressed in one or more of the developmental stages. Dunn’s multiple comparison post-test was used to detect at which developmental stages there were differences. Furthermore there was no evidence for Gaussian distribution in the datasets for 3’ UTR length analysis, including when they were converted to log values. Therefore a non-parametric Kruskal-Wallis test was also applied here to calculate if there were any differences in UTR length between different groups of transcripts. Dunn’s post test was applied to reveal which groups differed from each other. The statistical tests were performed using GraphPad Prism 5.04 (GraphPad Software Inc., La Jolla, CA92037, USA). A p-value of ≤0.05 indicates significant difference.

## Competing interests

The authors declare that they have no competing interests.

## Authors’ contributions

LK performed RNA isolation, qPCR analysis, *in silico* analysis of sequences that were mapped to the cod genome and drafted the manuscript. AW and GLT designed the study. AW and RBE sampled eggs/embryos and participated in the *in silico* analysis and manuscript preparation. AW designed the cDNA libraries for cod egg and developmental stages and HK and RR performed library sequencing. KM and TF carried out bioinformatical processing of sequence raw data. ØD provided guidance and participated in data analyses. All authors read and approved the final manuscript.

## Supplementary Material

Additional file 1 **Gene expression of reference genes in oocyte, 1-2 cell, blastula and gastrula stage in cod.** mRNA levels are shown as cycle threshold (Ct)-values when 250 ng RNA was used for cDNA synthesis. All data are shown as mean with SEM. N = 2. Click here for file

Additional file 2 **Gene expression levels of candidate genes to reveal timing of midblastula transition in cod.** mRNA levels of 3 candidate genes were measured in oocyte, 1-2 cell, blastula and gastrula stage. mRNA levels are relative to *hba*. Bars with different letters represent significant different mRNA levels for each gene. All data are shown as mean with SEM. N = 3. The maternal candidate chosen was the *eukaryotic translation initiation factor 4E* (*eif4e*) in which the 1b variant is found in oocyte and early embryo only in mouse (*eif4Elooeif41b*; [21]) and zebrafish [55]. Syntenic analysis in cod did not reveal *eif41b*, suggesting that this gene has been lost in this species (Data not shown, [27]). We measured *eif41a*, and mRNA levels significantly decreased from 1-2 cell stage to gastrula. In contrast, Robalino [57] detected a constant level of *eif41a* in oocyte and early embryo. *eif41a* is involved in recruiting cytoplasmic mRNA and initiation translation [58], and higher levels of *eif41a* generally correlate with increased protein synthesis and cell growth [59]. To indicate MZT, the *TATA box binding protein* (*tbp*) was selected. During early cleavage maternal stores of *tbp* are translated and by MBT the protein contribute to transcription initiation in *Xenopus*[60] and zebrafish [61]. *tbp* is also involved in degradation of maternal mRNAs [62], another main feature of MZT. In accordance with this, we detected a gradual decrease in *tbp* mRNA levels from oocyte until blastula. At gastrulation zygotic gene expression is presumably active, and one early zygotic gene in zebrafish is *no tail* (*t*), which is expressed in nuclei of the germ ring of the late blastula and early gastrula [63,64]. Likewise we did not detect expression of this gene until gastrula, where it was highly expressed. Click here for file

Additional file 3 **Distribution of nuclear genes with and without transcript number threshold.** The bar to the left represents the proportion of nuclear genes that were expressed exclusively in maternal stages, gastrula or in all four stages, when analyzing the dataset with a transcript number threshold (n > 8). The bar to the right represents the dataset when no threshold is applied. Total number of genes in the datasets (100%) is indicated on top of each bar. Click here for file

Additional file 4 **Functional profile of transcripts that showed a difference in abundance between maternal and zygotic stages.** Three levels of function (parent ontologies) are shown: cellular component, molecular function and biological process. The number on top of each bar represents the total number of transcripts in that stage, which could be coupled to the parent ontology (percentages are therefore calculated from these numbers as 100%). Each transcript may have multiple gene ontology annotations. Regarding cellular component 115 (oocyte), 122 (1-2 cell), 160 (blastula) and 80 (gastrula) transcripts did not have an annotation. The respective numbers for molecular function were 219, 250, 344 and 179, and for biological process they were 211, 251, 350 and 133. Macromol. comp. = macromolecular complex. Str. mol. act. = structural molecule activity. Cell. comp. org. = cellular component organization or biogenesis. Multicell. org. pr. = multicellular organismal process. Dev. pr. = developmental process. Click here for file

Additional file 5 **Results from repeated 3’UTR structure analyses, with less strict criteria for classification and excluding all ribosomal genes.** Results from repeated analyses on 3’UTR structure, with less strict classification of gene groups and excluding all ribosomal genes. In addition to genes with above 95% of transcripts present in oocyte until blastula stage (original analysis), genes with above 70% of transcripts present in oocyte and 1-2 cell were also included as degrading. Likewise, in addition to genes with above 95% of transcripts present in blastula and gastrula stage (original analysis), genes with above 70% of transcripts present in gastrula alone were also included as activating. Blastula stage may contain both maternal and zygotic transcripts; therefore including this stage in the less strict criteria for classification resulted in a number of genes that fell into more than 1 category. Therefore the less strict classification included transcripts of high proportion in oocyte and 1-2 cell or gastrula. For genes regarded as stable, the required proportion of transcripts in each of the four developmental stages (oocyte, 1-2 cell, blastula and gastrula) was extended from 25 ± 15% to 25 ± 20%. a) Overview of number of genes with 3’UTR isoforms (y-axis) within the whole dataset, and within genes with degrading, stable and activating transcripts (x-axis). The bar to the left within each category represents the total number of genes for that category, and the bar to the right represents the corresponding number of genes with 3’UTR isoforms. Percentages of genes with 3’UTR isoforms within each group are indicated by the number on top of the bars. b) 3’UTR-lengths of degrading, stable and activating transcripts. Different letters represent significant difference in 3’UTR length. All data are shown as mean with SEM. N = 69, 57 and 29 for degrading, stable and activating transcripts, respectively. c) Proportion (%) of degrading, stable and activating transcripts that contain different polyadenylation motifs (AATAAA, ATTAAA, AGTAAA, TATAAA, CATAAA, GATAAA, AATATA, AATACA, AATAGA, ACTAAA, AAGAAA and AATGAA). d) Proportion (%) of degrading, stable and activating transcripts that contain more than one of the motifs shown in c). N = 69, 57 and 29 for degrading, stable and activating transcripts, respectively. Click here for file

Additional file 6 **Contigs based on clustered sequences.** This file contains clustered sequences from all libraries, with given contig name. The contig name can be linked to annotation and accession number in [Additional file 7]. Click here for file

Additional file 7 **Contig annotations.** This file shows the mapping of EST-contigs to the Atlantic cod genome. Contig annotations for the 257 genes analysed in this study are shown. Click here for file

Additional file 8 **Gene Ontology annotation based on SwissProt BLAST hits.** This file contains Gene Ontology (GO) annotation based on SwissProt BLAST hits. 1) SwissProt gene symbol hit, 2) Ensembl gene, 3) Ensembl annotation, 4) SwissProt hit gene names, 5) SwissProt hit e-value, 6) SwissProt hit score, 7) Total SwissProt hits in the whole predicted gene set, 8) GO UniProtKB, 9) GO gene symbol, 10) GO number, 11) GO reference, 12) GO gene name, 13) GO alternative gene symbols, 14) Counter. Click here for file

Additional file 9 **Gene Ontology annotation based on UniRef90 BLAST hits.** This file contains Gene Ontology annotation based on UniRef90 BLAST hits. 1) UniRef hit, 2) Ensembl gene, 3) Ensembl annotation, 4) UniRef90 hit, 5) UniRef90 hit e-value, 6) UniRef90 hit score, 7) Total UniRef90 hits in the whole predicted gene set, 8) GO UniRef, 9) GO gene name, 10) GO number, 11), GO gene description, 12) Counter. Click here for file
