# Supplementary figures and images for: Maternal 3’UTRs: from egg to onset of zygotic transcription in Atlantic cod
Source: BMC Genomics. 2012 Sep 1;13:443. doi: 10.1186/1471-2164-13-443 (PMC3462720; doi:10.1186/1471-2164-13-443)

Mean Ct value

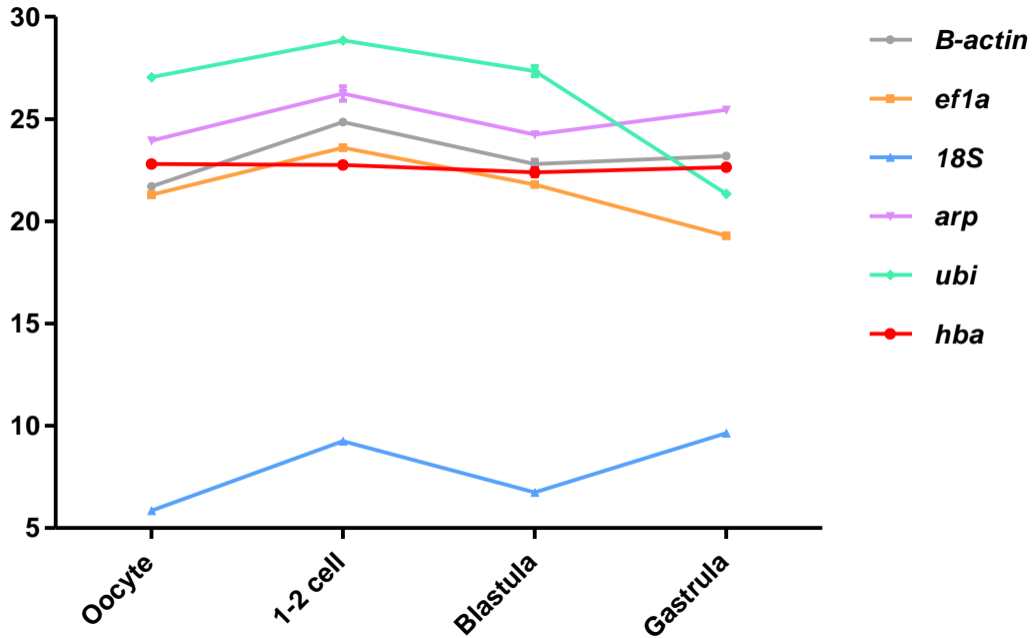

Supplement: Additional file 1 — Gene expression of reference genes in oocyte, 1-2 cell, blastula and gastrula stage in cod. mRNA levels are shown as cycle threshold (Ct)-values when 250 ng RNA was used for cDNA synthesis. All data are shown as mean with SEM. N = 2. [file 1471-2164-13-443-S1.pdf]

Relative gene expression

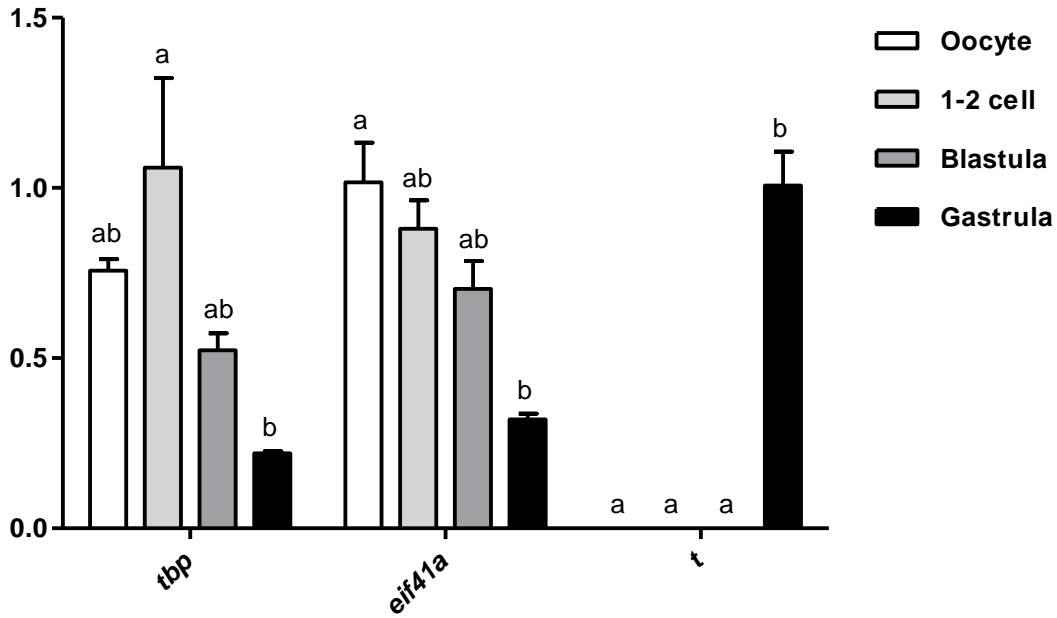

Supplement: Additional file 2 — Gene expression levels of candidate genes to reveal timing of midblastula transition in cod. mRNA levels of 3 candidate genes were measured in oocyte, 1-2 cell, blastula and gastrula stage. mRNA levels are relative to hba. Bars with different letters represent significant different mRNA levels for each gene. All data are shown as mean with SEM. N = 3. The maternal candidate chosen was the eukaryotic translation initiation factor 4E (eif4e) in which the 1b variant is found in oocyte and early embryo only in mouse (eif4Elooeif41b; [21]) and zebrafish [55]. Syntenic analysis in cod did not reveal eif41b, suggesting that this gene has been lost in this species (Data not shown, [27]). We measured eif41a, and mRNA levels significantly decreased from 1-2 cell stage to gastrula. In contrast, Robalino [57] detected a constant level of eif41a in oocyte and early embryo. eif41a is involved in recruiting cytoplasmic mRNA and initiation translation [58], and higher levels of eif41a generally correlate with increased protein synthesis and cell growth [59]. To indicate MZT, the TATA box binding protein (tbp) was selected. During early cleavage maternal stores of tbp are translated and by MBT the protein contribute to transcription initiation in Xenopus[60] and zebrafish [61]. tbp is also involved in degradation of maternal mRNAs [62], another main feature of MZT. In accordance with this, we detected a gradual decrease in tbp mRNA levels from oocyte until blastula. At gastrulation zygotic gene expression is presumably active, and one early zygotic gene in zebrafish is no tail (t), which is expressed in nuclei of the germ ring of the late blastula and early gastrula [63,64]. Likewise we did not detect expression of this gene until gastrula, where it was highly expressed. [file 1471-2164-13-443-S2.pdf]

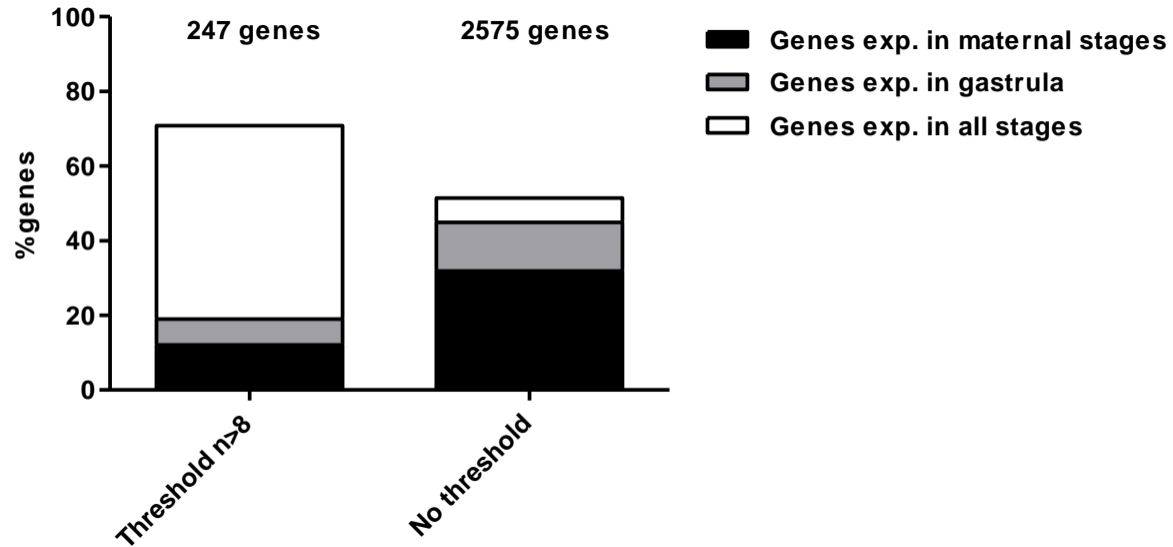

Supplement: Additional file 3 — Distribution of nuclear genes with and without transcript number threshold. The bar to the left represents the proportion of nuclear genes that were expressed exclusively in maternal stages, gastrula or in all four stages, when analyzing the dataset with a transcript number threshold (n > 8). The bar to the right represents the dataset when no threshold is applied. Total number of genes in the datasets (100%) is indicated on top of each bar. [file 1471-2164-13-443-S3.pdf]

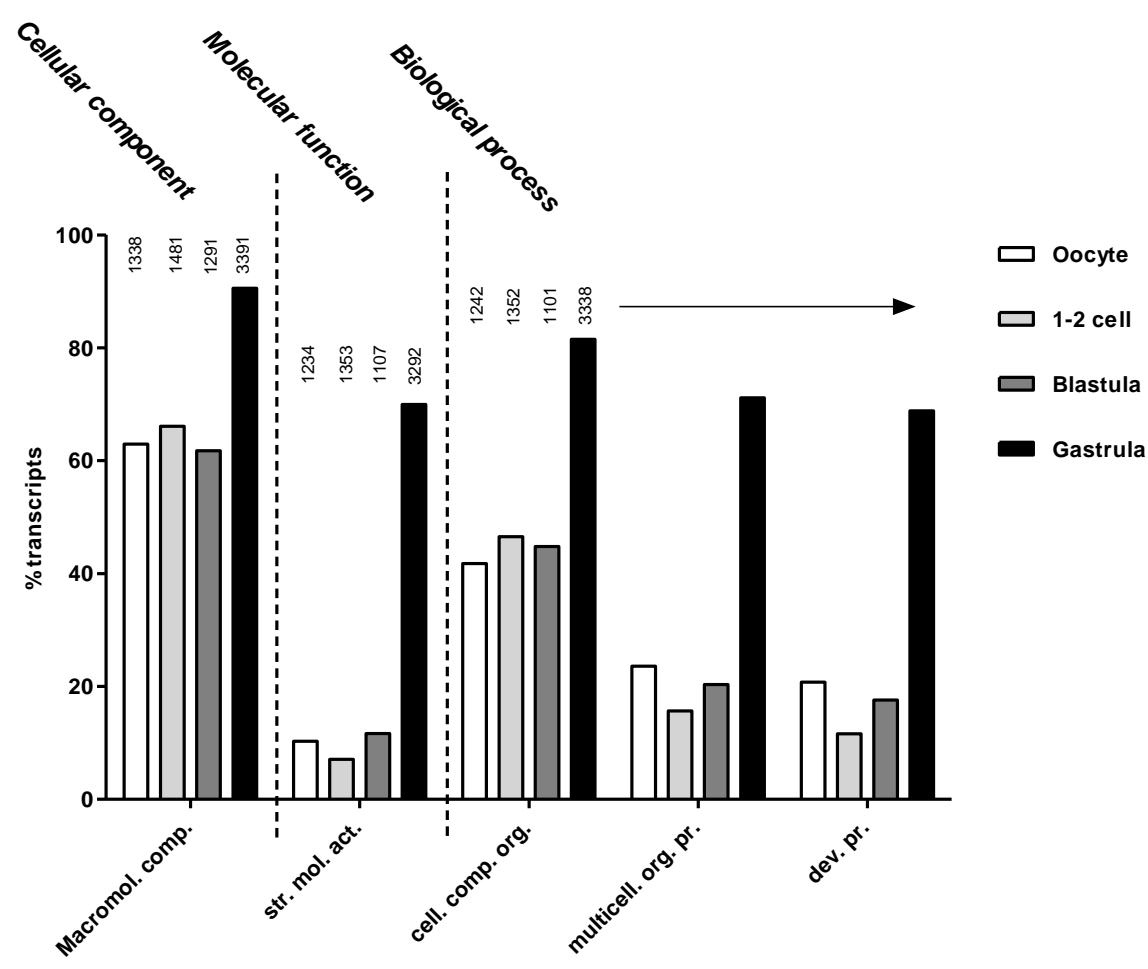

Supplement: Additional file 4 — Functional profile of transcripts that showed a difference in abundance between maternal and zygotic stages. Three levels of function (parent ontologies) are shown: cellular component, molecular function and biological process. The number on top of each bar represents the total number of transcripts in that stage, which could be coupled to the parent ontology (percentages are therefore calculated from these numbers as 100%). Each transcript may have multiple gene ontology annotations. Regarding cellular component 115 (oocyte), 122 (1-2 cell), 160 (blastula) and 80 (gastrula) transcripts did not have an annotation. The respective numbers for molecular function were 219, 250, 344 and 179, and for biological process they were 211, 251, 350 and 133. Macromol. comp. = macromolecular complex. Str. mol. act. = structural molecule activity. Cell. comp. org. = cellular component organization or biogenesis. Multicell. org. pr. = multicellular organismal process. Dev. pr. = developmental process. [file 1471-2164-13-443-S4.pdf]

a)

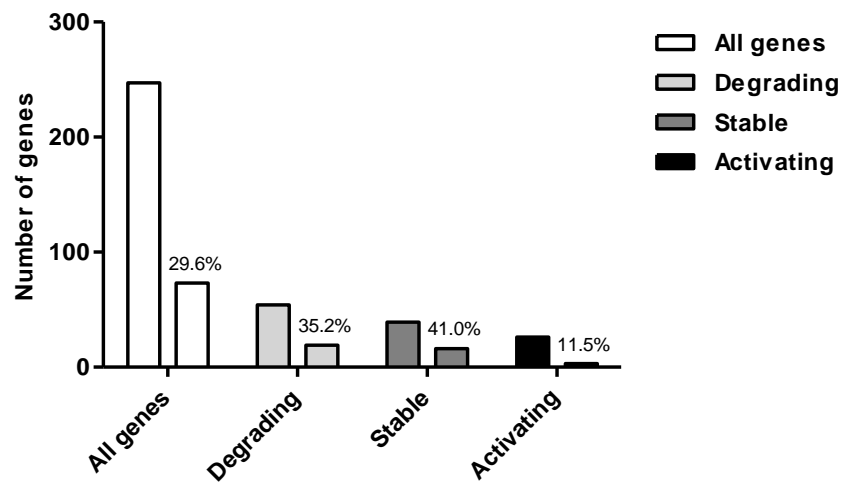

b)

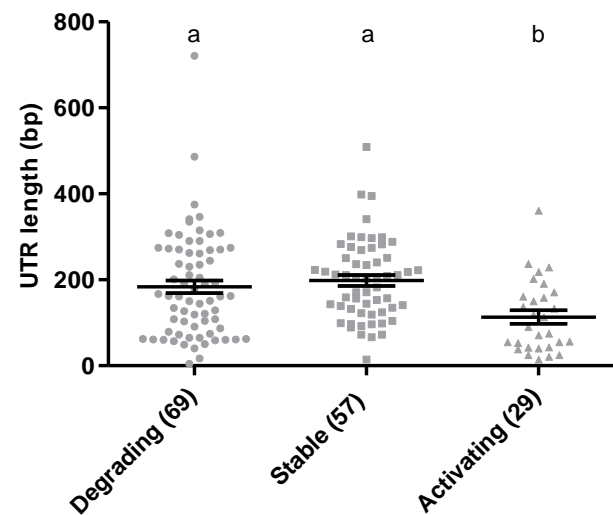

c)

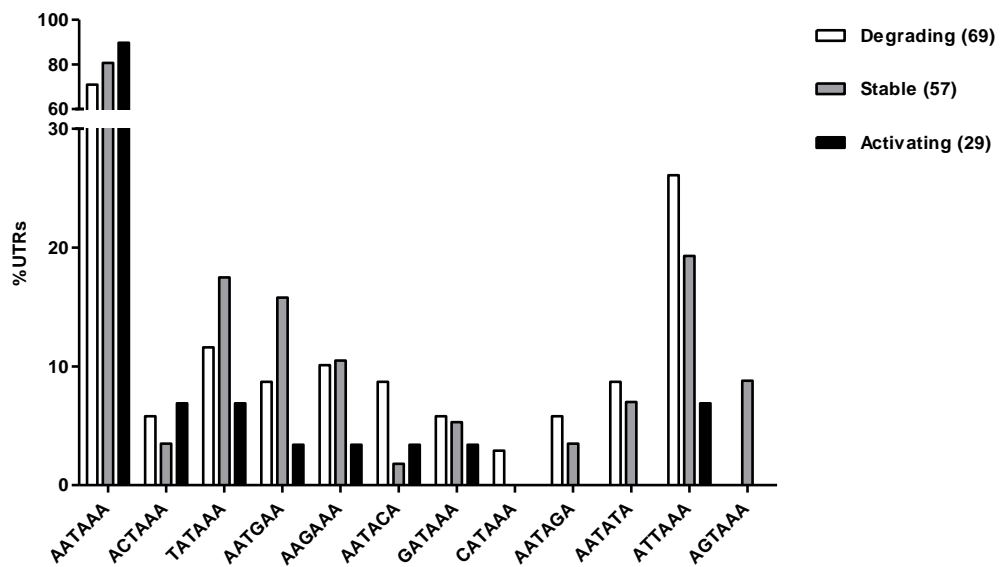

d)

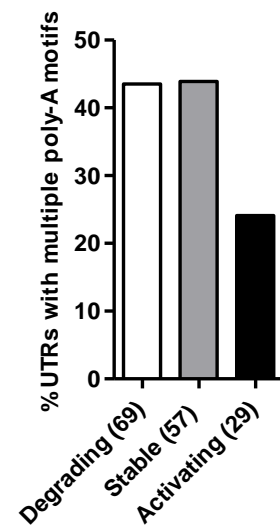

Supplement: Additional file 5 — Results from repeated 3’UTR structure analyses, with less strict criteria for classification and excluding all ribosomal genes. Results from repeated analyses on 3’UTR structure, with less strict classification of gene groups and excluding all ribosomal genes. In addition to genes with above 95% of transcripts present in oocyte until blastula stage (original analysis), genes with above 70% of transcripts present in oocyte and 1-2 cell were also included as degrading. Likewise, in addition to genes with above 95% of transcripts present in blastula and gastrula stage (original analysis), genes with above 70% of transcripts present in gastrula alone were also included as activating. Blastula stage may contain both maternal and zygotic transcripts; therefore including this stage in the less strict criteria for classification resulted in a number of genes that fell into more than 1 category. Therefore the less strict classification included transcripts of high proportion in oocyte and 1-2 cell or gastrula. For genes regarded as stable, the required proportion of transcripts in each of the four developmental stages (oocyte, 1-2 cell, blastula and gastrula) was extended from 25 ± 15% to 25 ± 20%. a) Overview of number of genes with 3’UTR isoforms (y-axis) within the whole dataset, and within genes with degrading, stable and activating transcripts (x-axis). The bar to the left within each category represents the total number of genes for that category, and the bar to the right represents the corresponding number of genes with 3’UTR isoforms. Percentages of genes with 3’UTR isoforms within each group are indicated by the number on top of the bars. b) 3’UTR-lengths of degrading, stable and activating transcripts. Different letters represent significant difference in 3’UTR length. All data are shown as mean with SEM. N = 69, 57 and 29 for degrading, stable and activating transcripts, respectively. c) Proportion (%) of degrading, stable and activating transcripts that contain d [file 1471-2164-13-443-S5.pdf]
